# Lost in Projection? Gaussian Filtering Recovers Hidden Conformational States

**DOI:** 10.1021/acs.jpclett.6c00341

**Published:** 2026-04-02

**Authors:** Sofia Sartore, Daniel Nagel, Georg Diez, Gerhard Stock

**Affiliations:** † Biomolecular Dynamics, Institute of Physics, 9174 University of Freiburg, 79104 Freiburg, Germany

## Abstract

To interpret molecular dynamics (MD) simulations, it is common practice to reduce the dimensionality of the molecular coordinates to a low-dimensional collective variable *x*. Projecting the high-dimensional MD data onto *x* yields a free energy landscape Δ*G*(*x*), which highlights low-energy regions corresponding to conformational states. The accurate definition of these states, however, is often impeded by projection artifacts, resulting in artificially shortened state lifetimes or even the complete disappearance of states from the analysis. As demonstrated for a two-dimensional toy model, Gaussian low-pass filtering of the high-dimensional feature trajectory can restore the underlying free energy landscape, allowing recovery of previously hidden states. When applied to an all-atom folding trajectory of HP35, the number of microstates increases by an order of magnitude, which leads to metastable states that are long-lived and much better defined structurally, even compared to dynamically cored state trajectories.

## Graphical abstract

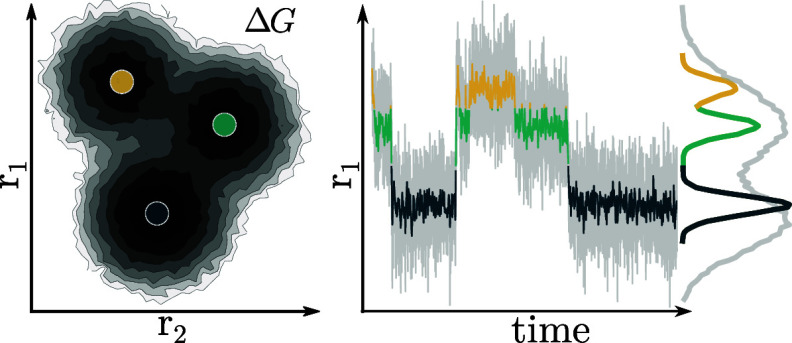

Complex dynamical systems, like solvated proteins, are characterized by dynamics that span multiple time scales. The output of molecular dynamics (MD) simulations provides the time evolution of the atomic coordinates, capturing fast local fluctuations and slower global conformational rearrangements.^1^ Since the latter usually constitute the processes of interest, the analysis of MD simulations involves separating fast and slow motions. This is often done by identifying metastable conformational states and modeling the dynamics of interest by a series of memoryless transitions between these states, yielding a Markov state model (MSM).^2–7^ This approach is widely used, since it holds the promise of predicting long-term dynamics from short trajectories and because its use is facilitated by workflow implementations in software packages like PyEmma,^8^ MSMBuilder,^9^ and msmhelper.^10^

Building meaningful MSMs from MD data, however, presents the challenge of correctly identifying metastable states. The high-dimensional space of atomic coordinates compromises this task at the very start, because the sparsity of the data distribution hinders statistical robustness. This issue can in principle be overcome by the assumption that protein dynamics reside on low-dimensional manifolds.^11,12^ All dimensionality reduction techniques, from straightforward linear principal component analysis^13^ to sophisticated deep-learning autoencoder strategies^14,15^ exploit this manifold hypothesis in order to project the initial coordinates onto a low-dimensional space of collective variables and effectively capture essential dynamics. The reduced-dimensional energy landscape of such projected coordinates is then scanned with a clustering algorithm that identifies the minima corresponding to metastable states. Density-based methods^16–19^ are a valuable alternative to the widely used geometric clusterings (such as *k*-means^20^) in MSM construction, as they perform better in defining states at the barriers.^21^ However, despite many advances in dimensionality reduction and clustering techniques, projecting high-dimensional data onto a lower-dimensional space often leads to misclassifications of data points, especially in sparsely populated transition regions. This hampers a proper definition of the states and their metastability, causing fast intrastate fluctuations to be interpreted as interstate transitions, which leads to an underestimation of the time scales of the processes under study. Even worse, it may also lead to the complete disappearance of states from the analysis.

A simple way of correcting these projection artifacts was introduced with the concept of coring.^3^ From a geometrical perspective, a core is defined as the region surrounding the center of a state. The idea is that the system should reach that region for a transition to a new state to be considered completed, thus resulting in a modified state trajectory.^22,23^ However, as the definition of geometrical cores is not always trivial, especially in the case of high dimensional coordinates, dynamical variants of coring were also introduced. These are generally based on the requirement that the system spends a minimum amount of time—the coring time *t*_cor_—in the new state after transitioning.^24,25^ Hence, *t*_cor_ poses a limit for the time resolution on the interstate dynamics and must therefore be chosen shorter than the fastest dynamics of interest.

Despite being effective in curing spurious interstate fluctuations, coring of the state trajectory cannot correct for the loss of states in the analysis, because coring is applied after the definition of states. This means that if the projection step causes the disappearance of free energy barriers (and thus the loss of states), coring of the state trajectory is not sufficient to recover the full dynamics. Recently, it has been shown that missing degrees of freedom can be recovered using time-aware representation learning methods exploiting temporal correlations.^26,27^ To introduce a data preprocessing step at the level of the input coordinates (or features) of the analysis, in this work we discuss ‘Gaussian filtering’ ^28,29^ that mitigates projection artifacts before clustering, by eliminating the high-frequency fluctuations in the coordinates trajectory. Acting as a low-pass filter, it is shown to improve the accuracy of state identification and subsequent MSM construction. While the use of low-pass filtering has been suggested before in the field of Markov modeling,^30–32^ we believe that its impact, utility, and consequences for MSM construction have not been fully appreciated so far.

We start with an intuitive example that reveals why artifacts are introduced by projecting data on a lower dimension and how the above-mentioned corrective approaches can offer promising solutions. The model consists of three potential wells separated by barriers of similar height (∼4 *k*_B_ *T*), see Figure 1. For this simple toy model, it is straightforward to define a true (i.e., optimal) reaction coordinate, *s*, following the lines connecting the minima,^33^ i.e., the segment between wells 1 and 2 for *x* ≤ 0 and the one between wells 2 and 3 for *x* > 0. We then run an overdamped Langevin simulation to sample the corresponding 2D free energy landscape (Figure 1a). As shown by the resulting 1D free energy curve Δ*G*(*s*) in Figure 1b, the three minima are clearly separated along *s*. The frames can be readily assigned to three states by cutting the curve exactly at the barriers, whose height is preserved from the 2D case. Figure 1c shows an example of the time trace of *s*, where the color of each frame represents the state it is assigned to. All three states are recovered and clearly separated, because along the true reaction coordinate the overdamped Langevin trajectory exhibits no immediate recrossing of the barrier after a transition, as it proceeds to the minimum of the new state. As a consequence, the state trajectories obtained from the 2D energy landscape Δ*G*(*x*, *y*) and the 1D landscape Δ*G*(*s*) are equivalent.

**Figure 1:**
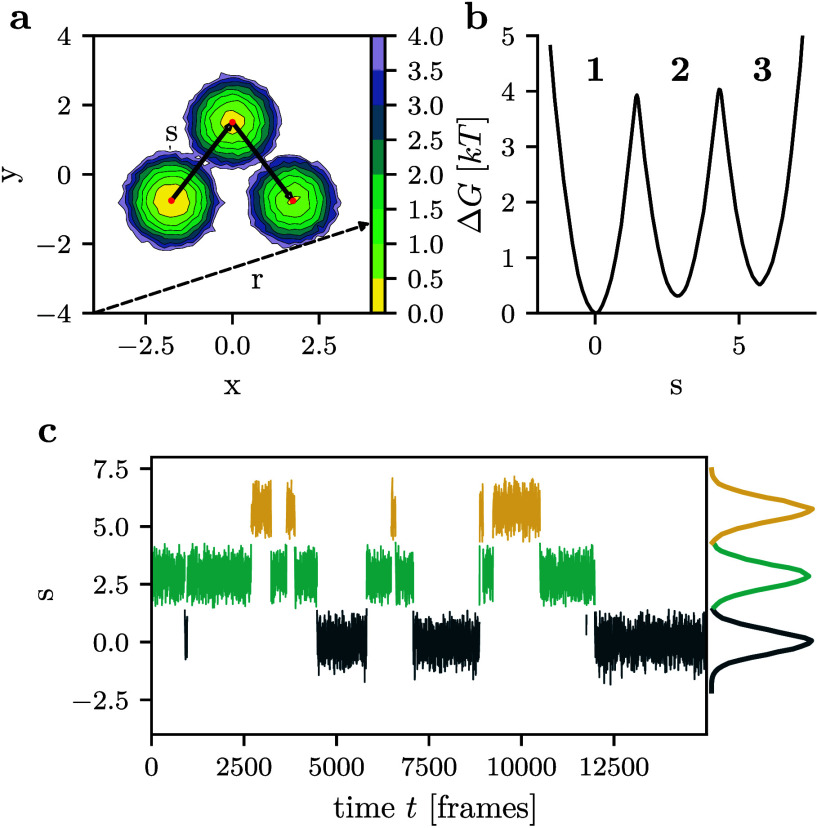
Three-well model. (a) 2D free energy landscape Δ*G*(*x*, *y*) as a function of the original coordinates *x* and *y*. Indicated are the optimal 1D coordinate, *s*, directly connecting the three minima, and a suboptimal coordinate, *r*, obtained via the projection on vector ***r***. (b) Projection of the 2D data on the optimal reaction coordinate *s* yields the barrier-preserving free energy curve Δ*G*(*s*). (c) Trajectory of the optimal reaction coordinate, color coded by the state that each frame is assigned to.

The situation becomes more involved in the—more realistic—case in which we only have a suboptimal reaction coordinate. To mimic this and illustrate how projection artifacts can arise in our toy model, we use *s* as a reference and define in Figure 1a two additional 1D reaction coordinates, where the projection effects become progressively worse: the projection on the *x*-axis ***x*** = *x* ***e***_*x*_, and the projection on the tilted axis ***r*** = *r* ***e***_*r*_.

As shown in Figure 2a, the three minima of Δ*G*(*x*) are still clearly distinguishable, but the barriers between them become smaller (∼1 *k*_B_ *T*) than in the original 2D landscape. Moreover, the time trace of *x* (Figure 2b) shows ‘overshooting’ at the barrier between states: transient fluctuations that are wrongly identified as transitions between states can be easily spotted as two different colors alternating very rapidly. This happens because frames located at the barriers of Δ*G*(*x*) may already belong to the adjacent state in the orthogonal *y*-direction. The loss of this information can be seen from the definition of the 1D free energy as

$$\Delta G\left(x\right)=-k_{B}T\;\ln P\left(x\right)$$ 1

with

P(x)=∫dyP(x,y) 2

When we consider the 2D probability distribution *P*(*x*_12_, *y*) at the barrier between states 1 and 2 along *x* (on the side of state 1), clearly the system may be already in state 2 along the *y* coordinate. As a result, the 1D trajectory *x*(*t*) exhibits the typical projection artifact of appearing to fluctuate rapidly back and forth across the barrier.

**Figure 2:**
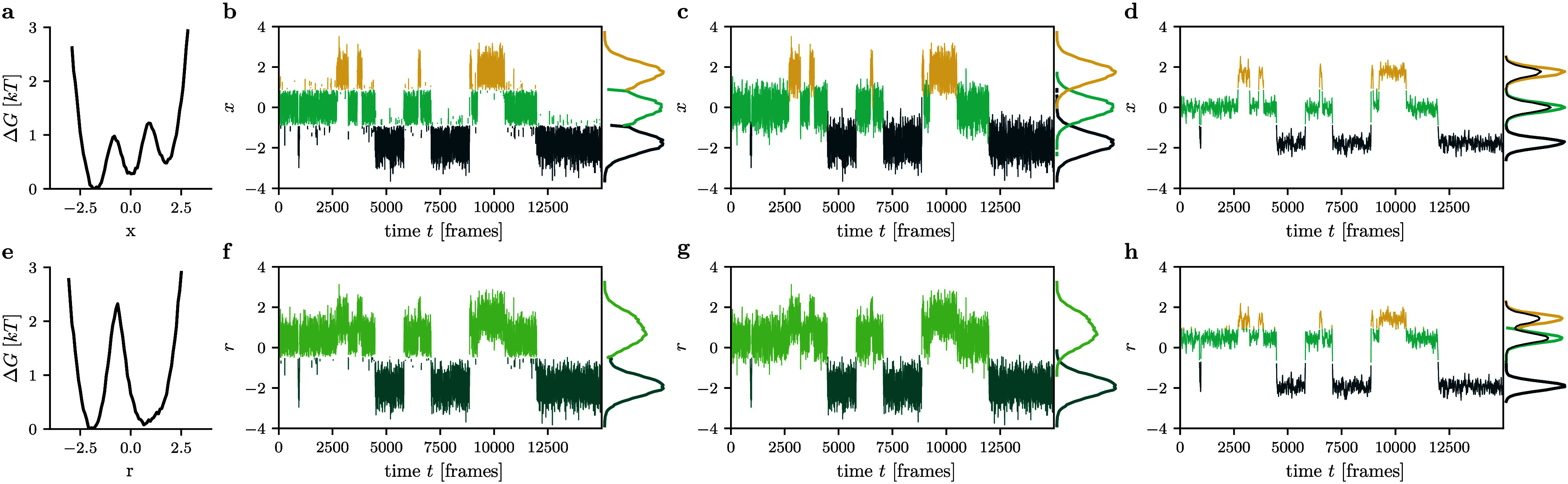
Effects of suboptimal 1D reaction coordinates *x* (top) and *r* (bottom) chosen for the 2D model. Shown are (left) 1D energy curves and (right) color-coded time traces with states assigned by cutting at the barrier. Panels b and f show the raw data, c and g the results after applying iterative coring (*t*_cor_ = 10 frames), and d and h the results after applying Gaussian filtering (*t*_GF_ = 10 frames). The right side of each panel shows the respective state-resolved distributions.

As a remedy, we apply dynamical coring;^24,25^ e.g., we request that the trajectory stays at least *t*_cor_ = 10 frames in the new state. As shown in Figure 2c, this simple measure already eliminates most of the spurious transitions at the barriers. In this way, the coring effectively reduces the overlap of the states’ probability distributions in the border regions. Since already small fluctuations may mask true transitions, it is in fact advantageous to iteratively core the state trajectory,^34^ by using coring times incrementing from *t*_cor_ = 2–10 frames, rather than performing a single coring process with *t*_cor_ = 10 frames. Interative coring is found to reduce the number of reassigned MD frames; i.e., it changes the original trajectory less, while still smoothing with the same time resolution.^35^

Alternatively, we may apply a Gaussian filter function with standard deviation σ on the feature trajectory *x*(*t*),^28,29^

$$x\left(t\right)\to \underset{j}{\sum }\frac{1}{\sqrt{2\pi \sigma^{2}}}\;\exp\left[-\frac{{\left(t_{j}-t\right)}^{2}}{2\sigma^{2}}\right]x\left(t_{j}\right)$$ 3

where *t*_*j*_ = *jδt*, with *δt* being the time step of the MD data, and *j* runs from −4σ/*δt* to +4σ/*δt*. It affects a smoothing of *x*(*t*) within a moving window of approximately *t*_GF_ = 2σ, corresponding to a low-pass filter with a cutoff frequency 1/*t*_GF_. Using *t*_GF_ = 10 frames, Figure 2d reveals that this smoothing results in a clear separation of the three energy basins. This is because Gaussian filtering causes a reduction of the amplitude of the fluctuations, which results in a narrowing of the distance distributions of the states. Hence, we achieve a better resolved structure of the free energy landscape with clearly defined barriers, before assigning the time series to states.

In the second, more drastic, case, we choose ***r*** = *r* ***e***_*r*_ (see Figure 1a) as projection axis for our model. The resulting free energy Δ*G*(*r*) (Figure 2e) reveals that one barrier is lost and only two states can be distinguished. Figure 2g shows the time-trace *r*(*t*) after applying iterative dynamical coring (*t*_cor_ = 10 frames) on the state trajectory. While the coring eliminates the fast fluctuations between the two states, it obviously cannot recover the information that is missing in the free energy landscape Δ*G*(*r*), as it can only reassign frames among the two states that were already identified. Nonetheless, the time evolution of *r*(*t*) still suggests the existence of three states (Figure 2f); hence, the question is how to retrieve this information at the level of the free energy.

Here the advantage of curing the problem at the level of the input coordinates is even more obvious, as can be seen in Figure 2h. By applying Gaussian filtering to *r*(*t*) with a short window of *t*_GF_ = 10 frames, the reduced fluctuations of *r*(*t*) facilitate the identification of all three minima of the free energy Δ*G*(*r*), therefore allowing us to assign the filtered data to three states.

To construct a MSM from these data, we compute the transition probabilities between all states within some lag time τ_lag_. Diagonalization of the resulting transition matrix yields the implied time scales (ITSs) for each value of τ_lag_, which can be used to assess how well an MSM reproduces the true dynamics of the system,^5^ see Figure 3. For the full 2D model, as well as in the case of the optimal reaction coordinate *s*, we find Markovian behavior, with ITSs independent of the lag time (black dashed lines). The slowest ITSs *t*_1_ reflects transitions between states 1 and 2 and state 3, while the second *t*_2_ captures the dynamics between state 2 and states 1 and 3.

**Figure 3:**
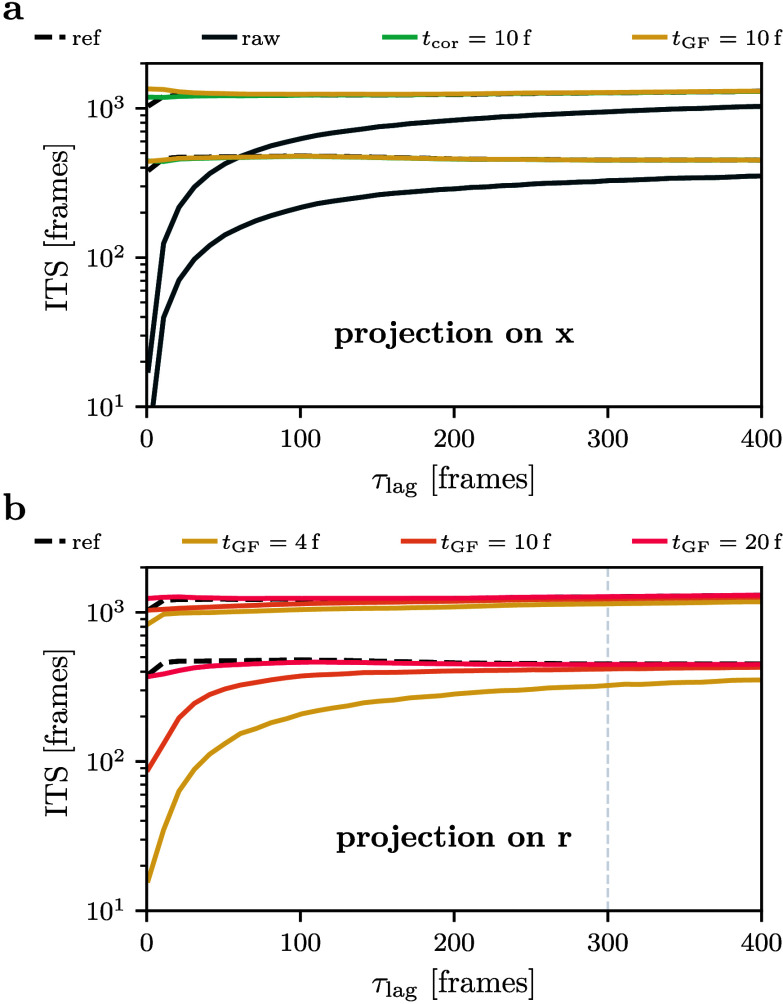
Implied time scales (ITSs) of the toy model, shown as a function of the lag time τ_lag_. (a) Projecting on coordinate *x*, we show uncorrected data and results from iterative coring (*t*_cor_ = 10 frames, green) and Gaussian filtering (*t*_GF_ = 10 frames, yellow). (b) Projecting on coordinate *r*, we use Gaussian filtering (*t*_GF_ = 4, 10, 20 frames) to recover both ITSs. In all cases we compare to the reference time scales obtained for the optimal coordinate *s* (dashed black lines).

Projecting on coordinate *x*, Figure 3a compares the results obtained for raw data (black), with coring on the state trajectory (green) and with Gaussian filtering on *x* (yellow). While ITSs obtained from the uncorrected data take a long lag time to converge to the reference results, both the coring and the Gaussian filtering results converge rapidly and reproduce the reference perfectly.

Projecting on coordinate *r*, uncorrected and cored data lead to a two-state model and therefore a single ITSs. Although coring again improves this ITSs, it cannot recover the third state. Gaussian filtering, on the other hand, is found to recover all three states for a filter window of *t*_GF_ ≳ 4 frames and yields a constant second ITSs for *t*_GF_ = 20 frames; see Figure 3b. The convergence of the implied time scales for increasing *t*_GF_ can be readily understood from the corresponding time traces shown in Figure S1 in the Supporting Information. For *t*_GF_ = 4 frames, we find (almost) no overshooting of *r*(*t*) out of state 1 (explaining that *t*_1_ is already converged), while there is still overshooting from state 2 to states 1 and 3. The latter disappears only for *t*_GF_ ≥ 20 frames, resulting in the convergence of *t*_2_.

Since both the coring time *t*_cor_ and the filtering window *t*_GF_ introduce a limit on the time resolution of the model, they need to be chosen shorter than the fastest time scale of interest. In particular, the lag time of an MSM built from cored and filtered data should be larger than *t*_cor_ and *t*_GF_, respectively. To preserve a good time resolution of the model, we therefore want to choose *t*_cor_ and *t*_GF_ as short as possible, while still correcting the projection error. As a heuristic to choose the coring time *t*_cor_, Nagel et al.^25^ suggested to compute the probability *W*_*i*_(*t*) of staying in macrostate *i* for various coring times and to choose the shortest *t*_cor_ that suppresses the rapid initial decay reflecting spurious transitions at the barrier. As a consequence, this procedure yields improved and sufficiently converged ITSs. Accordingly, in the case of Gaussian filtering we want to determine the shortest filtering window *t*_GF_ that yields sufficiently converged ITSs and, in addition, the correct number of states. From the above discussion of Figures 3 and Figure S1, we thus obtain *t*_GF_ = 10 and 20 frames for the projections on *x* and *r*, respectively.

We now study how the virtues of the above presented methods transfer to the treatment of high-dimensional MD data. To this end, we consider the folding of villin headpiece^36^ (Figure 4a), for which a 300 μs-long MD trajectory of the fast-folding Lys24Nle/Lys29Nle mutant (HP35) is publicly available from D. E. Shaw Research.^37^ In a first step we determined the inter-residue contacts of HP35, by assuming a contact to be formed if the distance *d*_*ij*_ between the closest non-hydrogen atoms of residues *i* and *j* is shorter than 4.5 Å, where |*i* – *j*| > 3 and *d*_*ij*_ is the minimal distance between all atom pairs of the two residues.^29,38^ To focus on native contacts, we request that contacts between these atoms pairs are populated for more than 30% of the simulation time,^29^ which results in 42 native contacts. Using MoSAIC correlation analysis,^39^ the associated contact distances are ordered in clusters of highly correlated contacts. Figure 4b shows the 27 contacts of the structurally most important clusters, which proceed along the protein backbone from the N- to the C-terminus. As a well-established 1D reaction coordinate,^40^ we evaluated along the trajectory the fraction of native contacts formed, *Q*(*t*), which nicely illustrates the time evolution of the folding process (Figure 4c).

**Figure 4:**
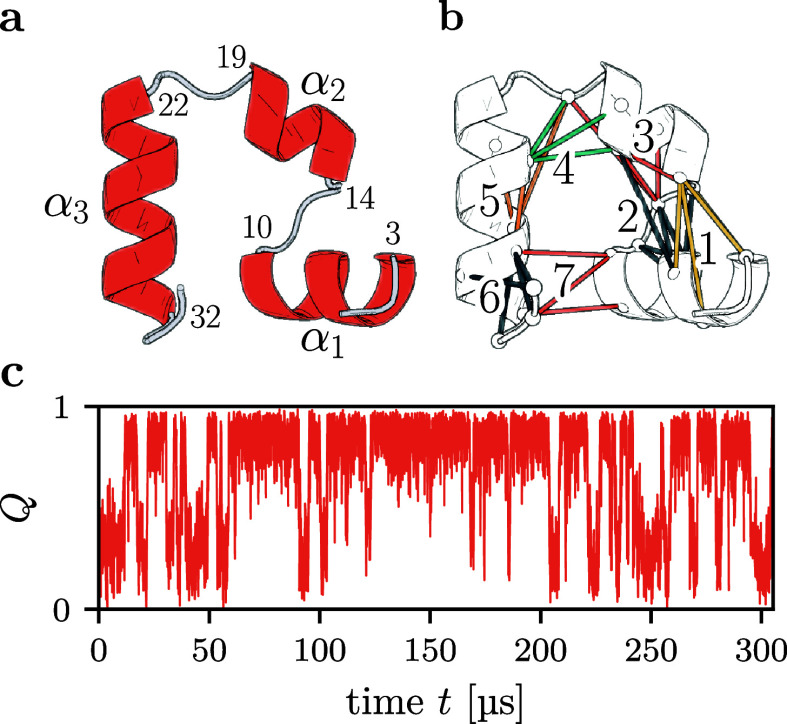
Folding of HP35. (a) Structure of the native state and (b) illustration of the structurally most important 27 native contacts, ordered in seven MoSAIC clusters.^39^ (c) Time evolution of the fraction of native contacts *Q* obtained from the folding trajectory by Piana et al.^37^ Adapted from ref 29.

To obtain a simple estimate of the folding time, we define the unfolded region of the free energy landscape by *Q* ≲ 0.3 and the native basin by *Q* ≳ 0.7, which allows us to directly count the folding events along the trajectory. Since *Q*(*t*) exhibits fast fluctuations that do not necessarily account for a true folding or unfolding event, we need to invoke a procedure to eliminate this noise. As for the 2D model studied above, we can either require a minimum time to stay in the new region (i.e., dynamical coring) or apply a low-pass filter to the data. In the following, we focus on the latter and employ Gaussian filtering of the contact distances used in the calculation of *Q*(*t*). We note in passing that we can also apply the filtering to Cartesian atom coordinates, from which the contact distances are calculated. While the outcomes of both approaches are quite similar (see Figure S2), we find that filtering the Cartesian coordinates may result in spurious fluctuations of the distances.^41^

Figure 5a shows the effects of Gaussian filtering on *Q*(*t*), by depicting the number of folding events as a function of the filtering window *t*_GF_ applied to the contacts. While the uncorrected data overestimate the number of folding events by a factor 2, we find that it takes *t*_GF_ ≳ 4 ns to converge to about 30 folding events. This corresponds to a folding time of ∼2 μs, which is in agreement with previous works.^37^ Yielding the correct folding time with minimal smoothing, *t*_GF_ = 4 ns was also chosen in the benchmark study by Nagel et al.^29^

**Figure 5:**
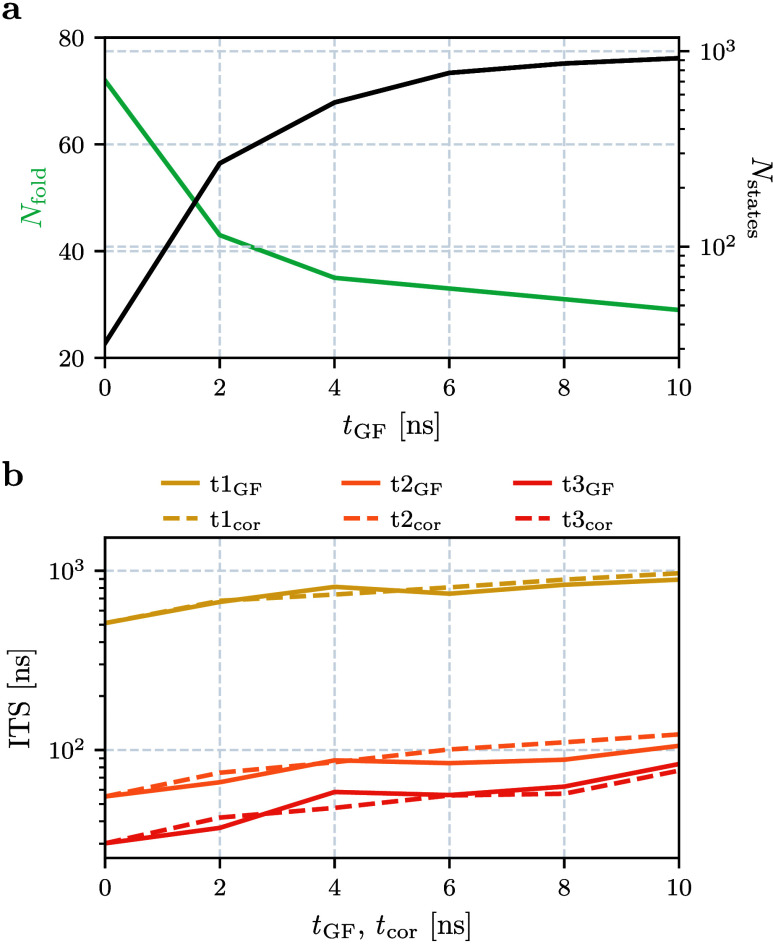
(a) Effects of Gaussian filtering with various filtering windows *t*_GF_ on the observed number of folding events *N*_fold_ of HP35 (green) and on the resulting number of microstates *N*_states_ obtained from robust density-based clustering^18^ (black). (b) First three implied time scales of MSMs obtained from coordinates filtered with different windows (full lines) and from dynamical coring on the microstates, using different coring times (dashed lines).

As discussed above, a major advantage of Gaussian filtering is that it can identify hidden conformational states, resulting in an improved MSM. To explore this, we use the 42 native contact distances of HP35 as input coordinates in the subsequent analysis and apply Gaussian filtering directly on these features. As dimensionality reduction step we use principal component analysis on the smoothed contact distances.^42^ The first five components exhibit a multimodal structure of their free energy curves, reveal the slowest time scales (∼0.1–2 μs), and explain ∼80% of the total correlation.^29^ Using these collective variables, we next perform robust density-based clustering.^18^ This clustering algorithm computes a local free energy estimate for every frame of the trajectory, by counting all other structures residing in a hypersphere of fixed radius. Reordering all structures by increasing free energy, the method directly yields the minima of the free energy landscape, where the number of these microstates depends on the chosen minimal population of a state (here *P*_min_ = 0.01%). (This is unlike the popular *k*-means clustering, where this number needs to be specified beforehand.)

Using different windows *t*_GF_ to filter the contact distances, the above workflow yields clusterings with different numbers of microstates *N*_states_. Remarkably, Figure 5a shows that *N*_states_ increases drastically with *t*_GF_, from 32 states for the uncorrected data (*t*_GF_ = 0), via 547 states (*t*_GF_ = 4 ns) to 990 states (*t*_GF_ = 10 ns), and stays approximately constant thereafter. This indicates that Gaussian filtering provides a significantly higher resolution of the underlying free energy landscape, confirming what we showed earlier for our toy model example.

To allow for a simple interpretation of an MSM, the numerous microstates are lumped into a few macrostates, which concisely account for the considered dynamical process. In this framework, differences between microstate partitionings do matter, because they may result in different macrostates and henceforth different dynamics. To group microstates into macrostates, we use the most probable path (MPP) algorithm,^24,43^ which constructs the transition matrix of the microstates and successively merges the state with the lowest metastability into the one to which it has the highest transition probability. Iteratively repeating this procedure yields a dendrogram that illustrates how the microstates aggregate into larger energy basins, which correspond to metastable conformational states. To resolve at least the first tier of the emerging hierarchical structure, we rely on the improved MPP algorithm^28^ that ensures that the states have a minimum metastability of 0.5 and a minimum population of 0.5%, thus obtaining 12 metastable states. Showing these dendrograms for (a) uncorrected and (b, c) Gaussian-filtered data with *t*_GF_ = 4 and 10 ns, Figure 6 clearly reveals the much larger number of microstates identified with Gaussian filtering. This leads to distinctly different lumping patterns and therefore also to different metastable states.

**Figure 6:**
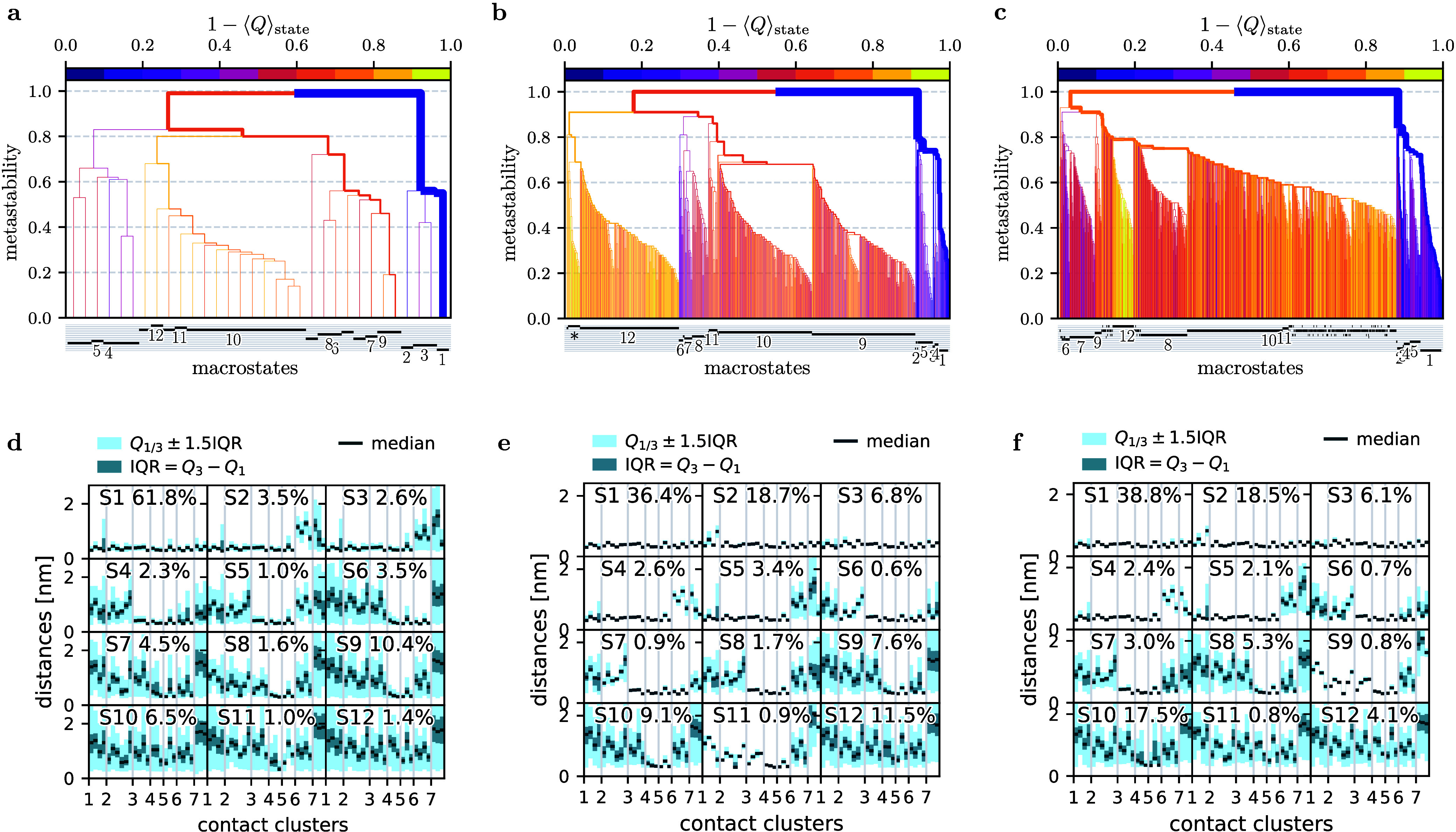
Construction of metastable conformational states describing the folding of HP35. Compared are uncorrected (a, d) data and Gaussian filtered data using (b, e) *t*_GF_ = 4 ns and (c, f) *t*_GF_ = 10 ns. (Top) MPP dendrogram showing the hierarchical clustering of microstates into 12 metastable macrostates, using a lag time of 10 ns. Colors indicate the fraction of native contacts *Q*. (Bottom) Structural characterization of the resulting 12 metastable states of HP35, ordered by decreasing *Q*. For each state, the distribution of contact distances are represented by the median *Q*_2_, the interquartile range IQR = *Q*_3_ – *Q*_1_ and the lower (upper) bound as the smallest (largest) data point in *Q*_1/3_ ± 1.5·IQR.

To illustrate this finding, Figures 6d–f present structural characterizations of the three resulting macrostate partitionings. For each state, the distributions of contact distances in MoSAIC clusters 1–7 (Figure 4b) are shown. The 12 macrostates are ordered by decreasing fractions of native contacts formed, such that state 1 corresponds to the native state (all distances shorter than 4.5 Å), whereas state 12 represents the fully unfolded state, with a broad distribution of large distances.

We first discuss the Gaussian-filtered data with *t*_GF_ = 4 ns (panel e) as used in the benchmark study by Nagel et al.^29^ and here referred to as ‘reference results’. The first three states are structurally well-defined, native-like conformations that differ mainly in the details of helix 1. States 4 and 5, which exhibit broken contacts near the C-terminus, still belong to the native energy basin. States 6–8 are sparsely populated (≲1%) intermediates, while the unfolded basin comprises states 9–12, showing an increasing degree of structural disorder. In particular, we find a completely unfolded state 12 and two largely unfolded states 9 and 10 (with mainly intact contacts between helices α_2_ and α_3_; see Figure 4b), which renders transitions from state 12 to states 9 and 10 the most likely initial step of the folding process.^28^

In contrast, the contact analysis of the uncorrected data (panel d) suggests that the first three states are merged into a single state. Obviously, the suppression of high-frequency fluctuations in the input data resulted in a better structural resolution of the native basin of HP35. Furthermore, the six states corresponding to the unfolded basin appear more congested and less distinctly separated than those obtained from the filtered reference data.

On the other hand, it is interesting to check if the state partitioning obtained for *t*_GF_ = 10 ns (where the number of microstates is maximal) presents an improvement over the reference results with *t*_GF_ = 4 ns. While the first six states are virtually the same, we find that states 7 and 8 of the reference are lumped into one state and that reference state 11 turns into state 9. The remaining unfolded states look similar to the reference state, but they do not clearly discriminate between the completely unfolded state and largely unfolded states with intact contacts between α_2_ and α_3_.

Apart from the structural properties of the macrostates, it is important to assess the dynamics of the various MSMs. To this end, Figure 5b shows their first three implied time scales (ITSs) as a function of the filtering window *t*_GF_, assuming a lag time of 10 ns. We find a clear increase of the first ITSs when comparing unfiltered (*t*_GF_ = 0) and reference (*t*_GF_ = 4 ns) results and a modest further improvement when using *t*_GF_ = 10 ns. Hence, Gaussian filtering improves the Markovianity of the model, by increasing the energy barriers due to a higher resolution of the free energy landscape, in particular in the transition state regions.

Following the discussion of the 2D model, we therefore conclude that *t*_GF_ = 4 ns used in the benchmark study by Nagel et al.^29^ represents the optimal choice of the filtering window. As shown in Figure 5, this yields good ITSs, the correct number of folding events *N*_fold_, and a significantly increased number of microstates *N*_states_. (Note that changing *t*_GF_ from 0 to 4 ns results in an increase of *N*_states_ by ∼1600%, while changing to 5 ns only gives a further increase of ∼80%.) Resulting in a time resolution of *t*_GF_ = 4 ns, this minimal size of the filtering window still allows using relatively short lag times in the construction of the MSM.

We finally compare the above MSMs obtained using Gaussian filtering of the input coordinates to MSMs obtained using iterative dynamical coring of the microstates. To this end, Figure 5b shows the first three ITSs as a function of the coring time *t*_cor_. Recalling that we need to choose *t*_cor_ ≈ *t*_GF_ to achieve a similar time resolution, we find that coring and filtering yield virtually the same time scales. However, as coring is applied to the microstates, it cannot achieve the improved structural resolution of the free energy landscape found for filtering (see Figure 6). In fact, the structural characterization of the macrostates obtained from coring (with *t*_cor_ = 4 ns) is virtually identical to the results obtained without coring shown in Figure 6d. In other words, dynamical coring only improves the dynamics of the resulting MSM, while Gaussian filtering improves in addition the structural properties of the macrostates of the model. Due to the complementary nature of the two methods, it may nevertheless be advantageous to use both corrections in order to optimize the Markovianity of the model.

In conclusion, we have shown that Gaussian filtering of input coordinates can significantly improve the performance of an MSM. Apart from naturally improving the time scale separation and therefore the implied time scales of the model, the reduction of the fluctuations may greatly facilitate the identification of microstates in the subsequent clustering of the data. We emphasize that this improved representation of the free energy landscape is only achieved because the filtering is applied at the very beginning of the trajectory analysis. In contrast, related approaches that correct for projection artifacts—such as dynamical coring of the state trajectory—cannot provide the same benefit. As the method is fully compatible with any subsequent workflow used to construct an MSM, we propose that initial Gaussian filtering of the feature trajectory should become a standard component of the MSM workflow.

Moreover we expect Gaussian filtering to be beneficial for a wide range of applications in MD analysis. For one, low-pass filtering improved the signal-to-noise ratio of time-dependent ensemble averages over nonequilibrium trajectories, revealing the allosteric transition in PDZ domains.^44^ As a further example, we mention change point detection,^45,46^ which aims at identifying abrupt changes in time-trace data, such as transitions between metastable conformations of the system. First results indicate indeed a significant increase in the robustness of the detected change points due to the removal of high-frequency noise.^27^

## Supplementary Material



## Data Availability

The simulation data and all intermediate results for our reference model of HP35, including our software packages *MoSAIC*,^39^ *FastPCA*,^47^ *Clustering* ^18^ and *msmhelper* ^10^ and detailed descriptions to reproduce all steps of the workflow can be downloaded from https://github.com/moldyn/HP35.

## Notes

Figure S1 showing the state trajectory and state distributions of the 2D model for various filtering times and Figure S2 comparing for HP35 the outcome of the direct filtering of features and the filtering of Cartesian atom coordinates to calculate features (PDF)

## ‡

Institut for Theoretical Physics, Heidelberg University, 69120 Heidelberg, Germany

## References

[1] Berendsen, H. J. Simulating the Physical World: Hierarchical Modeling from Quantum Mechanics to Fluid Dynamics; Cambridge University Press, 2007.

[2] Chodera J. D., Swope W. C., Pitera J. W., Dill K. A. (2006). Obtaining long-time
protein folding dynamics from short-time molecular dynamics simulations. Multiscale Modeling & Simulation.

[3] Buchete N.-V., Hummer G. (2008). Coarse master equations
for peptide folding dynamics. J. Phys. Chem.
B.

[4] Bowman G. R., Beauchamp K. A., Boxer G., Pande V. S. (2009). Progress and challenges
in the automated construction of Markov state models for full protein
systems. J. Chem. Phys..

[5] Prinz J.-H., Wu H., Sarich M., Keller B., Senne M., Held M., Chodera J. D., Schütte C. (2011). Markov models of molecular
kinetics: generation and validation. J. Chem.
Phys..

[6] Bowman, G. R. , Pande, V. S. , Noé, F. , Eds. An introduction to Markov state models and their application to long timescale molecular simulation; Advances in experimental medicine and biology 797; Springer, 2014.

[7] Wang W., Cao S., Zhu L., Huang X. (2018). Constructing Markov State Models
to elucidate the functional conformational changes of complex biomolecules. WIREs Comp. Mol. Sci..

[8] Scherer M. K., Trendelkamp-Schroer B., Paul F., Perez-Hernandez G., Hoffmann M., Plattner N., Wehmeyer C., Prinz J.-H. (2015). PyEMMA 2: A Software Package for Estimation, Validation, and Analysis
of Markov Models. J. Chem. Theory Comput..

[9] Beauchamp K. A., Bowman G. R., Lane T. J., Maibaum L., Haque I. S., Pande V. S. (2011). MSMBuilder2: Modeling
Conformational Dynamics on the
Picosecond to Millisecond Scale. J. Chem. Theory
Comput..

[10] Nagel D., Stock G. (2023). msmhelper: A Python
package for Markov state modeling of protein
dynamics. J. Open Source Softw..

[11] Hegger R., Altis A., Nguyen P. H., Stock G. (2007). How Complex is the
Dynamics of Peptide Folding?. Phys. Rev. Lett..

[12] Facco E., d’Errico M., Rodriguez A., Laio A. (2017). Estimating the intrinsic
dimension of datasets by a minimal neighborhood information. Sci. Rep..

[13] Amadei A., Linssen A. B. M., Berendsen H. J. C. (1993). Essential
dynamics of proteins. Proteins.

[14] Wang Y., Ribeiro J. M. L., Tiwary P. (2020). Machine learning
approaches for analyzing
and enhancing molecular dynamics simulations. Curr. Opin. Struct. Biol..

[15] Glielmo A., Husic B. E., Rodriguez A., Clementi C., Noé F., Laio A. (2021). Unsupervised Learning
Methods for Molecular Simulation Data. Chem.
Rev..

[16] Keller B., Daura X., van Gunsteren W. F. (2010). Comparing geometric and kinetic cluster
algorithms for molecular simulation data. J.
Chem. Phys..

[17] Rodriguez A., Laio A. (2014). Clustering
by fast search and find of density peaks. Science.

[18] Sittel F., Stock G. (2016). Robust Density-Based
Clustering to Identify Metastable Conformational
States of Proteins. J. Chem. Theory Comput..

[19] Liu S., Zhu L., Sheong F. K., Wang W., Huang X. (2017). Adaptive partitioning
by local density-peaks: An efficient density-based clustering algorithm
for analyzing molecular dynamics trajectories. J. Comput. Chem..

[20] Jain A. K. (2010). Data clustering:
50 years beyond K-means. Pattern Recognit. Lett..

[21] Sittel F., Stock G. (2018). Perspective: Identification
of Collective Coordinates and Metastable
States of Protein Dynamics. J. Chem. Phys..

[22] Schütte C., Noé F., Lu J., Sarich M., Vanden-Eijnden E. (2011). Markov state
models based on milestoning. J. Chem. Phys..

[23] Lemke O., Keller B. G. (2016). Density-based cluster
algorithms for the identification
of core sets. J. Chem. Phys..

[24] Jain A., Stock G. (2014). Hierarchical folding
free energy landscape of HP35 revealed by most
probable path clustering. J. Phys. Chem. B.

[25] Nagel D., Weber A., Lickert B., Stock G. (2019). Dynamical coring of
Markov state models. J. Chem. Phys..

[26] Wang D., Wang Y., Evans L., Tiwary P. (2024). From Latent Dynamics
to Meaningful Representations. J. Chem. Theory
Comput..

[27] Diez G., Dethloff N., Stock G. (2025). Recovering hidden degrees of freedom
using Gaussian processes. J. Chem. Phys..

[28] Nagel D., Sartore S., Stock G. (2023). Selecting
Features for Markov Modeling:
A Case Study on HP35. J. Chem. Theory Comput..

[29] Nagel D., Sartore S., Stock G. (2023). Toward a Benchmark
for Markov State
Models: The Folding of HP35. J. Phys. Chem.
Lett..

[30] Deng G., Cahill L. (1993). An adaptive Gaussian filter for noise reduction and
edge detection. 1993 IEEE Conference Record
Nuclear Science Symposium and Medical Imaging Conference.

[31] Jaipal M., Chatterjee A. (2017). Relative Occurrence
of Oxygen-Vacancy Pairs in Yttrium-Containing
Environments of Y2O3-Doped ZrO2 Can Be Crucial to Ionic Conductivity. J. Phys. Chem. C.

[32] Tan Z. W., Guarnera E., Berezovsky I. N. (2018). Exploring
chromatin hierarchical
organization via Markov State Modelling. PLOS
Comp. Bio..

[33] Krivov S. V. (2013). On Reaction
Coordinate Optimality. J. Chem. Theory Comput..

[34] Diez, G. Markov Modeling of Nonequilibrium Biomolecular Data. M.Sc. thesis, University of Freiburg, Germany, 2020.

[35] The improvement is modest for the simple 2D model: for *t* _cor_ = 10 frames standard and iterative coring reassign 5.14% vs 5.33% of the MD frames, respectively; for *t* _cor_ = 50 frames, though, we already find 5.29% vs 13.95%. For a complex system such as HP35 considered below, the effect is more substantial already at short coring times; e.g., we obtain 7.79% vs 11.57% for *t* _cor_ = 2 ns and 8.98% vs 15.03% for *t* _cor_ = 4 ns.

[36] Kubelka J., Henry E. R., Cellmer T., Hofrichter J., Eaton W. A. (2008). Chemical, physical, and theoretical
kinetics of an
ultrafast folding protein. Proc. Natl. Acad.
Sci. U.S.A..

[37] Piana S., Lindorff-Larsen K., Shaw D. E. (2012). Protein folding kinetics and thermodynamics
from atomistic simulation. Proc. Natl. Acad.
Sci. U.S.A..

[38] Yao X.-Q., Momin M., Hamelberg D. (2019). Establishing a Framework of Using
Residue–Residue Interactions in Protein Difference Network
Analysis. J. Chem. Inf. Model..

[39] Diez G., Nagel D., Stock G. (2022). Correlation-based
feature selection
to identify functional dynamics in proteins. J. Chem. Theory Comput..

[40] Best R. B., Hummer G., Eaton W. A. (2013). Native contacts
determine protein
folding mechanisms in atomistic simulations. Proc. Natl. Acad. Sci. U.S.A..

[41] This is related to fact that in one case we filter a distance (i.e., a single 1D variable), in the other case we separately filter two sets of coordinates (i.e., two independent 3D variables) that are also independently noisy. In the former case, various combinations of the positions of the two atoms may result in the same value for the distance, which is smoothed subsequently. Filtering the atoms’ coordinates separately, however, changes these positions and therefore results in general in different values for the distance compared to the first case.

[42] Ernst M., Sittel F., Stock G. (2015). Contact- and distance-based
principal
component analysis of protein dynamics. J. Chem.
Phys..

[43] Jain A., Stock G. (2012). Identifying metastable
states of folding proteins. J. Chem. Theory
Comput..

[44] Dorbath E., Rudolf F., Gulzar A., Stock G. (2026). Contact Cluster Modeling
of Allosteric Communication in PDZ Domains. J. Phys. Chem. B.

[45] Truong C., Oudre L., Vayatis N. (2020). Selective review of
offline change
point detection methods. Signal Process..

[46] Romano G., Rigaill G., Runge V., Fearnhead P. (2022). Detecting
abrupt changes in the presence of local fluctuations and autocorrelated
noise. J. Am. Stat. Assoc..

[47] Sittel F., Filk T., Stock G. (2017). Principal
component analysis on a
torus: Theory and application to protein dynamics. J. Chem. Phys..

