# Supplementary material for: Lost in Projection? Gaussian Filtering Recovers Hidden Conformational States
Source: J Phys Chem Lett. 2026 Apr 2;17(15):4418–25. doi: 10.1021/acs.jpclett.6c00341 (PMC13093668; doi:10.1021/acs.jpclett.6c00341)
Supplement: Supplementary file 1 [file jz6c00341_si_001.pdf]

Supporting Information: Lost in Projection? Gaussian Filtering Recovers Hidden Conformational States

Sofia Sartore,[†,¶] Daniel Nagel,[†,‡,¶] Georg Diez,[†] and Gerhard Stock[*,†]

†*Biomolecular Dynamics, Institute of Physics, University of Freiburg, 79104 Freiburg, Germany*
‡*current address: Institut for Theoretical Physics, Heidelberg University, 69120 Heidelberg, Germany*
¶*Contributed equally to this work*

E-mail: stock@physik.uni-freiburg.de

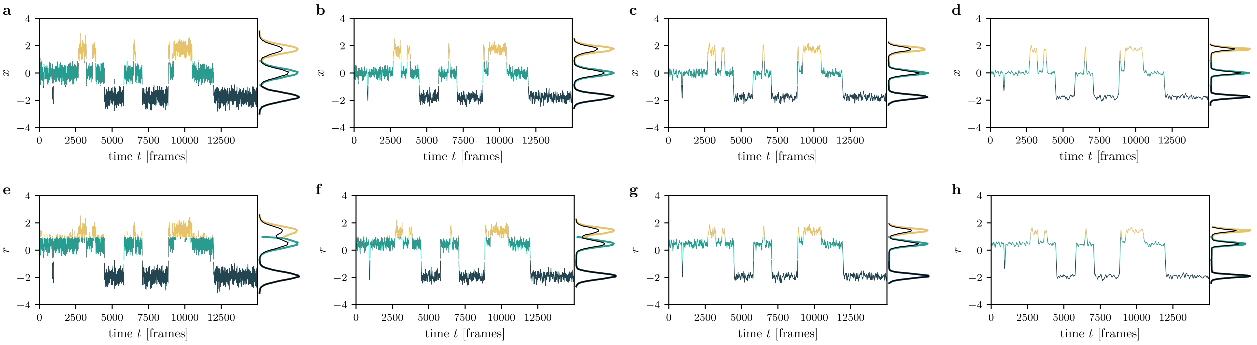

Figure S1: Effects of suboptimal 1D reaction coordinates x (top) and r (bottom) chosen for the 2D model presented in Figs. 1 and 2 of the main text. Shown are color-coded time traces with states assigned by cutting at the barrier after applying Gaussian filtering of increasing window. We show the effect of $t_{\mathrm{GF}} = 4$ frames (a, e), $t_{\mathrm{GF}} = 10$ frames (b, f), $t_{\mathrm{GF}} = 20$ frames (c, g) and $t_{\mathrm{GF}} = 50$ frames (d, h). The right side of each panel shows the respective state-resolved distributions.

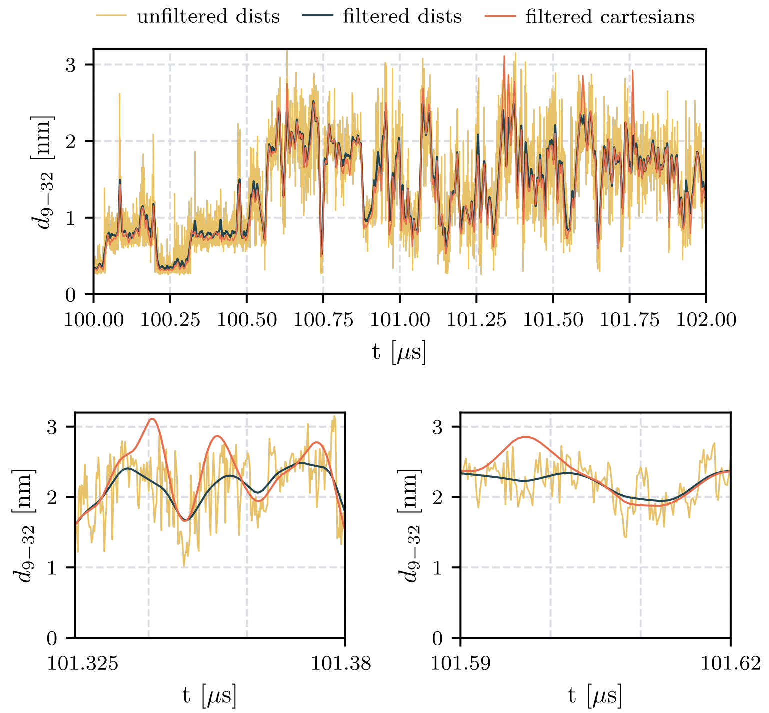

Figure S2: Example of the time evolution of a representative contact distance of HP35, using Gaussian filtering ($t_{\mathrm{GF}} = 4$ ns) on the contact distances (dark green) and on the original Cartesian coordinates of the atoms (orange). Both resulting distance are plotted over the original unfiltered distance (yellow) for a $2\,\mu$s long fragment of the total trajectory (upper panel). The lower panels show two zoom-ins as examples of the different results yielded by the two filtering approaches.
